# Use of Social Media as a Tool to Reduce Antibiotic Usage: A Neglected Approach to Combat Antimicrobial Resistance in Low and Middle Income Countries

**DOI:** 10.3389/fpubh.2020.558576

**Published:** 2020-12-10

**Authors:** Krishna Prasad Acharya, Deepak Subedi

**Affiliations:** ^1^Animal Quarantine Office (AQO), Kathmandu, Nepal; ^2^Institute of Agriculture and Animal Sciences (IAAS), Tribhuvan University (TU), Paklihawa, Rupandehi, Nepal

**Keywords:** social media, tool, use, antimicrobial resistance (AMR), low and middle income countries (LMICs)

The burden of antimicrobial resistance (AMR) is continuously growing, especially among low- and middle-income countries (LMICs). Irrational and excessive use of antimicrobials has been the leading cause of this in these countries. Many of the strategies that were recommended to tackle AMR included following infection prevention and control (IPCs) measures and improving hygiene and sanitation standards, as well as prudent antibiotic use with no/minimal antibiotics application (1, 2). Nonetheless, in most LMICs, the rate of implementation of such initiatives is very low. Prudent medical use of antimicrobials and following recommended drug medication schedule (strict adherence to recommended guidelines) by the consumers; proper infection prevention and control measures; and the incorporation of appropriate guidelines on the rational use of antibiotics into clinical practice have potential to decrease the pace of development of AMR and could prevent the development of resistant microbes (3, 4). An estimated 10 Million deaths a year due to antimicrobial resistance by 2050 (5) reflects the risk associated with it and shows need for different tools to combat it.

In these days of technological advancement, consumers and users of antimicrobials could be sensitized and made aware of by the effective use of social media like YouTube, Facebook, Twitter, online games etc. (6) to make people aware of concepts of AMR and its effect on human, animal and environmental health. Over 3.5 billion people (47% of the global population) use the internet via mobile devices, which are the major means of internet and social media in LMICs (7). The increase in the use of mobiles and social media in LMICs is an opportunity to increase awareness about disease prevention, and rational use of antibiotic and antibiotic resistance via various online videos, games and images. The use of social media for health benefits has been demonstrated in various research conducted in high income countries (e.g., United States and United Kingdom). Social media has the capacity to deliver health education, communicate useful information, and help in the coordination of health workers in LMICs (8). Dissemination of information via social media is faster and cheaper than traditional methods and can reach larger audiences in short period of time. In addition, its use can be easily adopted according to target population and time frame.

The Centers for Disease Control and Prevention (CDC) is attempting to raise the antibiotic awareness using different social media (9). Various types of online games could be created with a serious learning objective, and these games could be used to disseminate necessary information and learning about AMR to the kids while they play them. These games could be designed to run on smartphones. In the same way, some puzzles regarding antibiotics (their use, side effects, and benefits) could be created to be disseminated by public authorities at the school level. Attempt had been made to increase antibiotic awareness among school students in the UK via online games by an education resource called “e-Bug” -this resulted in significant increase in knowledge for two out of seven questions and two of the e-Bug antibiotic educational games were found to be valuable (10). The antimicrobial stewardship program has also used social media like Facebook and Twitter to increase awareness among internal medicine residents (11).

Furthermore, physicians and all other healthcare professionals working in both primary and secondary healthcare settings in middle and low-income countries could be trained using mobile phones apps in the best use of antibiotics as they are significant prescribers of medicines (12). To enhance this impact, awareness seminars should be arranged at the public and professional level on a regular basis. Similarly, school-level teachers should be given a useful training on related matters via a similar route (e.g., maintaining proper sanitation and hygiene, good infection control and prevention measures, and appropriate and rational use of antibiotics). This would have an impact on information they impart to their pupils and hence have long-lasting impacts. These techniques could potentially reduce the unjustified use of antimicrobials, and so help counter the use of antibiotics to compensate for poor hygiene and sanitation in LMICs (a widespread practice). These issues should be introduced and incorporated into the guidelines and action plans in LMICs.

Up to now, studies have mainly concentrated on the rational use of antibiotics, focused upon physicians and patients, with only occasionally dispersed, unorganized, and inconsistent awareness campaigns. Sustained attempts to raise awareness of this crucial issue of public concern has been missing and including AMR issues into social media platforms could be an effective dissemination vehicle. This inclusion of AMR issues into social media could be a cornerstone in the worldwide fight against AMR if thoroughly tested in field conditions and used at a larger scale in the best public interests.

Once educated, health care professionals and general public at different levels could easily convince their fellow members concerning these issues to reduce the burden of resistant microbes by the overall minimization of the use of antibiotics (13, 14). This minimization of the use of antibiotics could effectively reduce the AMR in the long term, as seen in Sweden and Finland (15, 16). As a result of this, the overall burden of resistant microbes and AMR could be reduced, and the overall well-being of humans and all other living beings be promoted.

## Author Contributions

KA conceived the idea and wrote the initial manuscript. KA and DS extensively revised the manuscript and approved for final publication.

## Conflict of Interest

The authors declare that the research was conducted in the absence of any commercial or financial relationships that could be construed as a potential conflict of interest.
